# Fulminant type 1 diabetes developed after influenza split vaccination

**DOI:** 10.1530/EDM-22-0342

**Published:** 2023-05-08

**Authors:** Toshitaka Sawamura, Shigehiro Karashima, Ai Ohmori, Kei Sawada, Daisuke Aono, Mitsuhiro Kometani, Yoshiyu Takeda, Takashi Yoneda

**Affiliations:** 1 Department of Internal Medicine, Asanogawa General Hospital, Kosakamachinaka, Kanazawa, Ishikawa, Japan; 2 Department of Endocrinology and Metabolism, Kanazawa University Graduate School of Medicine, Takaramachi, Kanazawa, Japan; 3 Department of Health Promotion and Medicine of the Future, Kanazawa University, Takaramachi, Kanazawa, Japan

**Keywords:** Adult, Male, Asian - Japanese, Japan, Pancreas, Diabetes, Unique/unexpected symptoms or presentations of a disease, May, 2023

## Abstract

**Summary:**

Fulminant type 1 diabetes (FT1D) is a subtype of diabetes characterized by rapid progression of β-cell destruction, hyperglycemia, and diabetic ketoacidosis (DKA). The pathogenesis of this disease remains unclear. However, viral infections, HLA genes, and immune checkpoint inhibitor use were reportedly involved in this disease. A 51-year-old Japanese man with no chronic medical condition was admitted to our hospital with complaints of nausea and vomiting. Cough, sore throat, nasal discharge, and diarrhea were not noted. He had a medical history of at least two influenza infections. His vaccination history was notable for receiving an inactive split influenza vaccine 12 days prior to developing these symptoms. He was diagnosed with DKA associated with FT1D. His HLA class II genotypes were nonsusceptible to FT1D, and he had a negative history of immune checkpoint inhibitor use. The destruction of the pancreas by cytotoxic T cells is reported to be involved in FT1D. Inactive split influenza vaccines do not directly activate cytotoxic T cells. However, these could activate the redifferentiation of memory CD8-positive T cells into cytotoxic T cells and induce FT1D, as this patient had a history of influenza infections.

**Learning points:**

- Influenza split vaccination could cause fulminant type 1 diabetes (FT1D).
- The mechanism of influenza split vaccine-induced FT1D might be through the redifferentiation of CD8-positive memory T cells into cytotoxic T cells.

## Background

Fulminant type 1 diabetes (FT1D) is a subtype of type 1 diabetes characterized by rapid progression of β-cell destruction, hyperglycemia, and diabetic ketoacidosis (DKA) within a few days. The diagnostic criteria described by Imagawa *et al.* include the following: (1) rapid progression to DKA or ketosis, (2) plasma glucose level higher than 288 mg/dL, (3) hemoglobin A1c (HbA1c) level lower than 8.7%, and (4) fasting serum C-peptide level lower than 0.3 ng/mL with C-peptide level lower than 0.5 ng/mL after glucagon loading (1).

FT1D lacks the insulitis and hyperexpression of MCH class I in the islet cells. Compared with other types of type 1 diabetes, the onset that is dramatic and almost no β-cells remain at disease onset. In addition to β-cell, the destruction of α-cell and cellular infiltration in the exocrine tissue are also observed in FT1D. Cellular immunity is thought to be involved, and islet-related antibodies are usually negative. Moreover, HLA DRB1*0405-DQB1*0401 is reported as a susceptible haplotype (1, 2, 3).

It has been reported that the activation of autoimmunity as a history of viral infections, certain HLA polymorphisms, and an administration of immune checkpoint inhibitors contribute to the development of FT1D (2).

Here, we describe a case of FT1D developed after inactive split influenza vaccination, in the absence of classic triggers.

## Case presentation

A 51-year-old Japanese man with no chronic medical conditions presented to our hospital with complaints of nausea and vomiting for 2 days. He complained of polyuria and polydipsia for 5 days prior to admission. Cough, sore throat, nasal discharge, and diarrhea were not noted. His medical history was notable for 2 prior influenza infections about 20 years ago and 5 years ago. His family history was unremarkable. Moreover, he did not have a cold, acute hepatitis, shingles, herpes infection, or diarrhea by rotavirus within one year. He was not taking any medications. His fasting plasma glucose level was 101 mg/dL, and his HbA1c level was 5.8% in the health check-up performed 40 days prior to admission. His vaccination history was notable for receiving an inactive split influenza vaccine 12 days prior to developing these symptoms. He had not received any other vaccines in the preceding year.

On admission, his body weight and BMI were 69.0 kg and 25.9 kg/m^2^, respectively. His body weight decreased 2.0 kg in past 1 week. He had a blood pressure of 101/70 mmHg, a heart rate of 133 beats/min, a temperature of 37.1 ℃, a oxygen saturation of 98% in room air, and a respiratory rate of 36 breaths/min. There were no abnormalities upon examining the chest and abdomen.

## Investigation

As shown in Table 1, laboratory evaluation revealed high blood glucose level (779 mg/dL), metabolic acidosis, elevated serum total ketone level, mildly elevated HbA1c level (7.6%), and markedly elevated glycoalbumin level (37.4%). Moreover, an elevation in serum amylase level (215 mg/dL) was observed. Evaluation of C-peptide immunoreactivity indicated an insulin-insufficient state. He was diagnosed with DKA associated with FT1D. Anti-glutamic acid decarboxylase antibody, anti-insulinoma-associated antigen-2 antibody, islet cell antibody, and anti-zinc transporter 8 antibody were all negative. His HLA class II genotypes were DRB1*15:02-DQB1*06:01 and DRB1*04;06-DQB1*06:01, which are nonsusceptible to FT1D.

**Table 1:** Laboratory examination on admission.

	Value	Normal range
Blood		
WBC count, /μL	24 600	4800–10 800
RBC count, × 10^6^/μL	5.75	4.20–5.40
Hemoglobin, g/dL	19.1	12.0–16.0
Platelet count, × 10^3^/μL	292	130–400
AST, U/L	16	13–33
ALT, U/L	32	6–222
Total bilirubin, mg/dL	0.8	0.4–1.2
Amylase, mg/dL	215	37–124
Total protein, g/dL	7.4	6.7–8.3
Albumin, g/dL	4.7	3.8–5.2
Sodium, mEq/L	129	135–149
Potassium, mEq/L	5.5	3.5–4.9
Chloride, mEq/L	88	96–108
BUN, mg/dL	46	8.0–22.0
Creatinine, mg/dL	1.72	0.50–0.80
CRP, mg/dL	0.23	<0.3
Glucose, mg/dL	779	69–140
HbA1c, %	7.6	4.6–6.2
GA, %	37.4	12.4–16.3
CPR, ng/mL	0.26	0.8–2.5
Total ketone body, μmol/L	8443	26–122
Acetoacelate, μmol/L	2681	13–69
3-Hydroxybutyrate, μmol/L	5762	0–76
GAD antibody	Negative	Negative
IA-2 antibody	Negative	Negative
ICA antibody	Negative	Negative
ZnT8 zntibody	Negative	Negative
Urine		
Specific gravity	1.037	1.005–1.030
Glucose	4+	Negative
Protein	Negative	Negative
Ketone body	4+	Negative
Urine C-peptide, μg/day	2.3	29.2–167.0
Arterial blood gas		
PH	7.189	7.38–7.46
pCO_2_, mmHg	15.7	32.0–46.0
pO_2_, mmHg	136.7	74.0–108.0
HCO3^−^, mEq/L	5.8	21.0–29.0
BE, mEq/L	−19.2	−2 to 2

ALT, alanine aminotransferase; AST, aspartate aminotransferase; BE, base excess; BUN, blood urea nitrogen; CPR, C-peptide immunoreactivity; CRP, C-reactive protein; GA, glycoalbumin; GAD, anti-glutamic acid decarboxylase; HbA1c, hemoglobin A1c; IA-2, Insulinoma-associated antigen-2; IC, islet cell; pH, potential hydrogen; RBC, red blood cell; WBC, white blood cell; ZnT8, zinc transporter 8.

## Treatment

Intravenous insulin and saline infusions were administered, leading to an improvement in his symptoms, blood glucose level, and metabolic acidosis. After 2 days of i.v. insulin therapy, he was started on a diet with s.c. multiple daily injections (MDI), which included insulin glargine U300 and insulin lispro. The course of blood glucose levels, serum amylase levels, and C-peptide immunoreactivity is described in Table 2. Finally, the patient was discharged with an insulin daily total dose of 46 units, which were 0.66 units/kg at day 15. His body weight returned to 71.2 kg at the time of discharge.

**Table 2:** The course of insulin secretion and blood parameters.

	Health check-up	On admission	Day 3	Day 7	Day 14	6 Months
Amylase, U/L	N/A	215	176	74	56	63
CPR, ng/mL	N/A	0.26	<0.03	N/A	<0.03	<0.03
BG, mg/dL	101	779	131	124	111	105
HbA1c, %	5.8	7.6	N/A	N/A	N/A	7.4
A/G ratio	1.41	1.74	1.74	1.37	1.45	1.33

A/G ratio, albumin to globulin ratio; BG, blood glucose level; CPR, C-peptide immunoreactivity; HbA1c, hemoglobin A1c.

## Outcome and follow-up

After his discharge, treatment with multiple daily injection (MDI) and diet therapy was continued. Six months after his discharge, his HbA1c level was 7.4% and his total daily insulin dose was 40 units.

## Discussion

This was a case of FT1D developed after inactive split influenza vaccination.

Shibasaki *et al.* reported the expression of Toll-like receptor-7 (TLR-7) and cytotoxic T cells invasion in the pancreas, in the autopsies of patients with FT1D (4). Moreover, Chujo *et al.* revealed that CD8-positive cytotoxic T cells were more common in patients with FT1D than in patients with other type 1 diabetes by an integrated assay using peripheral blood mononuclear cells (5). Therefore, cytotoxic T cells-induced destruction of the pancreas is the most plausible mechanism of FT1D.

Sano *et al.* reported the first case of FT1D after influenza B virus infection (6). The patient had no remarkable medical and family histories. Moreover, the HLA gene genotype of this patient was DRB1*0405-DQB1*0401. FT1D was developed 6 days after the influenza B virus infection. Although the trigger is different and the HLA gene genotype is susceptible to FT1D, this case is comparable to our case in terms of the onset time from the trigger and clinical course. Nishioka *et al.* reported a 1.3-fold increase in the incidence of FT1D within 180 days after influenza infection (7). When a patient is infected with the influenza virus, several pathogen recognition receptors including the TLR-7 recognize the single binding RNA of the influenza virus. The recognition of the virus by dendritic cells through TLR-7 results in the activation of cytotoxic T cells (8). With a strongly activated immune system, TLR-7 recognizes not only the single binding RNA of the virus but also could recognize human own cells as the target for attack. Therefore, cytotoxic T cells destroy the pancreas, and FT1D can occur after viral infections.

There was only one report in which FT1D after influenza split vaccination was described. In this report, FT1D was developed 7 days after the vaccination and combined with thrombocytopenia and lymphopenia (9). However, a clear mechanism of FT1D in this case was not proven.

Koyama *et al.* showed that inactive split vaccine does not directly activate cellular immunity through TLR-7 in mice (10). This is because the TLR-7 ligand is lost during the production process. Therefore, split influenza vaccination alone is unlikely to cause FT1D through the TLR-7 pathway. However, our patient has a history of influenza infections. After the influenza virus infection, parts of activated cytotoxic T cells differentiate into memory CD8-positive T cells (11). At the time of the next influenza infection or vaccination, these CD8-positive memory T cells can re-differentiate into cytotoxic T cells and initiate the immune response. In this patient, redifferentiation into cytotoxic T cells after the influenza split vaccination could destroy the pancreas and induce FT1D.

We also examined the involvement of humoral immunity. Four known antibodies were all negative. Moldoneanu *et al.* reported that a raise in serum IgG level was seen after 13 days from the vaccination (12). In our case, the symptoms related to diabetes were seen after 7 days from the vaccination. If the destruction of the pancreas was caused by humoral immunity, this onset time is considered too early. The serum albumin to globulin ratio reflects the activation of humoral immunity. In our case, the serum albumin to globulin ratio was unchanged between vaccination and 6 months. These findings indicated that it is unlikely that humoral immunity was associated with the destruction of the pancreas.

We described a case of FT1D developed after influenza split vaccination. Considering the results of previous studies, the redifferentiation of memory CD8-positive T cells into cytotoxic T cells is the most likely mechanism of pathogenesis. Influenza vaccination is widespread and commonly used in daily practice. It is important to accumulate case reports to clarify the mechanism and frequency of FT1D after the influenza vaccination.

## Declaration of interest

The authors declare that no conflict of interest could be perceived as prejudicing the impartiality of the research reported.

## Funding

This work did not receive any specific grant from any funding agency in the public, commercial, or not-for-profit sector.

## Patient consent

Written informed consent for publication of clinical details was obtained from the patient.

## Patient’s perspective

I was very worried because after the influenza vaccine was administered, I began to have severe physical discomfort, which gradually worsened. I know that side effects are an inevitable part of medical treatment, but I think there are others like me, so I hope that I can be of some help to medicine by sharing information.

## Author contribution statement

TS was the attending physician of this patient. AO, KS, and YT worked with the attending physician to provide medical care for this patient during his hospitalization. TS wrote the manuscript. SK supervised manuscript writing. DA, MK, and TY were medical advisers of the Endocrine and Diabetes Unit of Kanazawa University Hospital. All authors have read and agreed to the published version of the manuscript.
